# Chikungunya Virus-associated Long-term Arthralgia: A 36-month Prospective Longitudinal Study

**DOI:** 10.1371/journal.pntd.0002137

**Published:** 2013-03-21

**Authors:** Clémentine Schilte, Frédérik Staikovsky, Thérèse Couderc, Yoann Madec, Florence Carpentier, Somar Kassab, Matthew L. Albert, Marc Lecuit, Alain Michault

**Affiliations:** 1 Unité Immunobiologie des Cellules Dendritiques, Department of Immunology, Institut Pasteur, Paris, France; 2 Institut National de la Santé et de la Recherche Médicale (INSERM) U818, Paris, France; 3 Emergency Department, Pôle des Spécialités de l'Urgence, Centre Hospitalier Régional de La Réunion, Groupe Hospitalier Sud Réunion, Saint Pierre, La Réunion, France; 4 Biology of Infection Unit, Institut Pasteur, Paris, France; 5 INSERM U1117, Paris, France; 6 Unité d'Epidémiologie des maladies émergentes, Institut Pasteur, Paris, France; 7 AgroParisTech, Ecologie Adaptation et Interaction, Paris, France; 8 INRA, UR1290, Thiverval-Grignon, France; 9 Université Paris Descartes, Sorbonne Paris Cité, Paris, France; 10 Division of Infectious Diseases, Hôpital Necker Enfants malades, Paris, France; 11 Department of Microbiology, Pôle de biologie, Centre Hospitalier Régional de La Réunion, Groupe Hospitalier Sud Réunion, Saint Pierre, La Réunion, France; Centre for Cellular and Molecular Biology, India

## Abstract

**Background:**

Arthritogenic alphaviruses, including Chikungunya virus (CHIKV), are responsible for acute fever and arthralgia, but can also lead to chronic symptoms. In 2006, a Chikungunya outbreak occurred in La Réunion Island, during which we constituted a prospective cohort of viremic patients (n = 180) and defined the clinical and biological features of acute infection. Individuals were followed as part of a longitudinal study to investigate in details the long-term outcome of Chikungunya.

**Methodology/Principal Findings:**

Patients were submitted to clinical investigations 4, 6, 14 and 36 months after presentation with acute CHIKV infection. At 36 months, 22 patients with arthralgia and 20 patients without arthralgia were randomly selected from the cohort and consented for blood sampling. During the 3 years following acute infection, 60% of patients had experienced symptoms of arthralgia, with most reporting episodic relapse and recovery periods. Long-term arthralgias were typically polyarthralgia (70%), that were usually symmetrical (90%) and highly incapacitating (77%). They were often associated with local swelling (63%), asthenia (77%) or depression (56%). The age over 35 years and the presence of arthralgia 4 months after the disease onset are risk factors of long-term arthralgia. Patients with long-term arthralgia did not display biological markers typically found in autoimmune or rheumatoid diseases. These data helped define the features of CHIKV-associated chronic arthralgia and permitted an estimation of the economic burden associated with arthralgia.

**Conclusions/Significance:**

This study demonstrates that chronic arthralgia is a frequent complication of acute Chikungunya disease and suggests that it results from a local rather than systemic inflammation.

## Author Summary

Chikungunya virus (CHIKV) is transmitted to human by mosquitoes. It is a re-emerging virus that has a risk to spread globally, given the expanding dissemination of its mosquito vectors. Chikungunya disease is characterized by acute transient febrile arthralgic illness, but can also lead to chronic incapacitating arthralgia. We have conducted a prospective longitudinal study to investigate in details long-term outcome of CHIKV infection. We found that 60% of patients experienced arthralgia 36 months after the onset of acute disease. Arthralgia affected most often multiple sites and were usually incapacitating. In addition to arthralgia, many patients suffered from myalgia and cutaneous lesions and several cognitive dysfunctions. We also showed that age over 35 years and the presence of arthralgia 4 months after the onset of disease are risk factors for long-term arthralgia. Patients with long-term arthralgia did not display biological markers typically found in autoimmune or rheumatoid diseases. This study demonstrates that chronic arthralgia is a frequent complication of acute Chikungunya disease and suggests that it results from a local rather than systemic inflammation.

## Introduction

Chikungunya virus (CHIKV) is an arthropod-borne virus that belongs to the *Alphavirus* genus. Chikungunya disease is characterized by polyarthralgia, sometimes associated with rash. The articular symptoms, often debilitating, usually resolve within weeks, but have been reported to last for months, even though the natural history of this infection has not been precisely studied in prospective studies [1], [2], [3].

In 2005, CHIKV emerged in islands of Indian Ocean including La Réunion, a French overseas department, and approximately one third of the inhabitants (*i.e.* ∼300,000) was infected at the end of the outbreak in 2006 [4], [5]. Compared to earlier outbreaks, this episode occurred in a highly medicalized area. Moreover previously unreported severe forms of Chikungunya were observed, such as encephalopathy [6], [7], and mother-to-child CHIKV transmission was demonstrated, leading to severe neonatal CHIKV infection [5]. In the wake of this outbreak, CHIKV also re-emerged in India with over 1 million cases [8], [9]. In 2007, CHIKV emerged for the first time in Europe, causing an outbreak in Italy [10].

We have described the clinical and biological features of acute CHIKV infection in a prospective cohort of patients with positive blood CHIKV RT-PCR [11]. It included all patients referred to the Emergency Department in Saint-Pierre de la Réunion with febrile arthralgia between March and May 2006. As little is known about long-term outcome of CHIKV infection, we conducted a prospective longitudinal study to describe in details the specific clinical and biological features of chronic arthralgia, as well as clinical signs associated to this pathology. We evaluated the consequences of long-term arthralgia on patients' daily and social life, looked for risk factors associated with them and estimated their economic impact.

## Materials and Methods

### Ethics & STROBE statement

Ethical clearance was obtained from the «Comité de Protection des Personnes Sud-Ouest et Outre-Mer III» of Saint-Pierre, La Réunion, Paris (CCP 2008/65, n° 2008-A00999-46). CHIK-IMMUNOPATH received approval from the ethical committee for studies with human subjects (CPP) of Bordeaux and the National Commission for Informatics and Liberty (CNIL). Written informed consent was obtained from patients included in the CHIK-IMMUNOPATH study. The study respects the STROBE statement (supporting information S1).

### Patients

We studied a cohort of patients (n = 180) enrolled for febrile arthralgia to the Emergency Department of the Groupe Hospitalier Sud Réunion between March 2005 and May 2006 [11]. Patients were interviewed by telephone 4, 6, 14 and 36 months (M4, M6, M14 and M36) after the viremic phase, using the same questionnaire as that used at day 0 (D0) (supporting information S2). At M36 after the acute phase, all patients who agreed to participate to a complementary study (CHIK-IMMUNOPATH) and who were arthralgic were interviewed and underwent clinical examination. Among them, 22 patients with arthralgia (ART+) and 20 patients without persisting arthralgia (ART−) were randomly selected from the cohort. They signed a written consent and a blood sample was collected.

### Laboratory tests performed at M36

Blood cell count was performed and viremia was tested by qRT-PCR [12]. Both serum anti-CHIKV IgG and IgM specific antibodies were screened. An enzyme-linked immunosorbent assay (ELISA) was performed with CHIKV antigen [12]. The avidity of anti-CHIKV IgG was tested by ELISA in the presence or absence of urea 8 M [13]. Geometric Mean Antibody Titer (GMAT) was calculated as previously described [14].

Plasmatic protein electrophoresis was performed and C-reactive protein (CRP) concentration was measured. The presence of anti-nuclear, anti-dsDNA, anti-endomysium autoantibodies, anti-cyclic citrullinated peptide antibody (ACCP) and cryoglobulinemia were investigated. Samples were transported at 37°C to research cryoglobulins. Sera were sent to Myriad RBM (Austin,Texas) and analyzed by Luminex using the inflammation MAP. Assays are run according to CLIA guidelines and in all cases, >100 beads per analyte were measured with CV <10% for values that are above the limit of quantification for the given assay.

### Statistical analysis

For each time point, the proportion of patients with monoarthralgia (1 site), oligoarthralgia (2–3 sites) or polyarthralgia (4 sites or more) were compared using a Chi-2 test.

A logistic regression model was used to identify factors associated with long-term arthralgia, defined as presence of arthralgia at M14. We studied factors at D0, including demographic factors (gender and age), biological markers, hospitalization and comorbidities, and factors measured at M4 (arthralgia, treatment, and quality of life). All factors associated with long-term arthralgia with a p-value<0.15 in univariate analysis were entered in the multivariate model. A step-by-step backward procedure was then used to identify factors significantly associated with long-term arthralgia. A sensitivity analysis was also conducted, following the same procedure, defining long-term persistence of arthralgia as the presence of arthralgia at M36. To address the lost of follow up at M36, we looked for parameters that differentiate the patients who were lost between M14 and M36 and these who were followed.

Statistical analyses were performed using the STATA software (Stata Corporation, College Station, Texas, USA); all significance tests were two-sided and p-values<0.05 were considered significant. Luminex data were mined using the Omniwiz software (Biowisdom) and Mann-Whitney analysis is reported. False discovery rate (FDR or *q-*values) were calculated as correction for multiple analyte testing.

To characterize the spatiotemporal evolution of arthralgia, we considered different types of arthralgia. For a given site at a given time point, arthralgia was defined either as a “persistent symptom” if the affected site was the same as that reported during the previous time point, as “a relapse of an the acute symptom” if the site affected was the same as that at the acute phase, or as “a new symptom” if this site was not affected at the acute phase, or as a “migrating symptom” if the arthralgia was localized at a site distinct from that previously reported. For a given patient, these categories were not mutually exclusive. For modeling migratory arthralgia, we divided joints into three groups: upper limb joints (hand, wrist, elbow), mid body joints, (shoulder, spine, hip) and lower limb joints (foot, ankle, knee), and considered two migratory probabilities for migration to sites within to the same group (e.g., hand to wrist) or to a different group (e.g., hand to foot) (Supporting information S3 and Table S1).

## Results

### Characteristics of long-term arthralgia in CHIKV-infected patients

**Figure 1:**
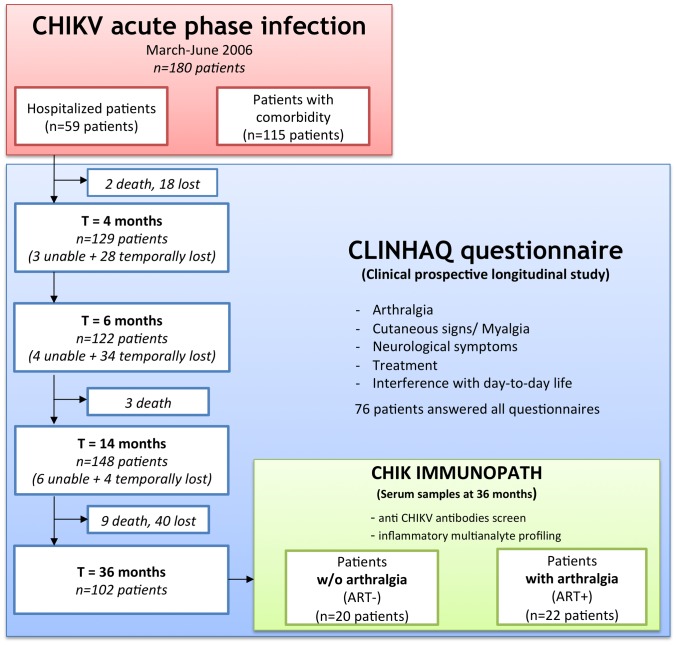
Diagram of the clinical study. “unable”: patients who were reached but unable to answer to the questionnaire, “lost”: patient lost of follow up until the end of the study, “temporally lost”: not reached at a specific timepoint, “death”: dead patients.

At M4, M6, M14 and M36 after their inclusion as acute CHIKV-infected patients, all patients were interviewed using a questionnaire to monitor persistence of arthralgia, other clinical signs and treatments. The number of patients that participated is provided in Figure 1. There is an important lost of follow up between M14 and M36 however we did not identify a bias associated with it. Among the 180 patients, 76 patients were followed at all time points of the study.

**Figure 2:**
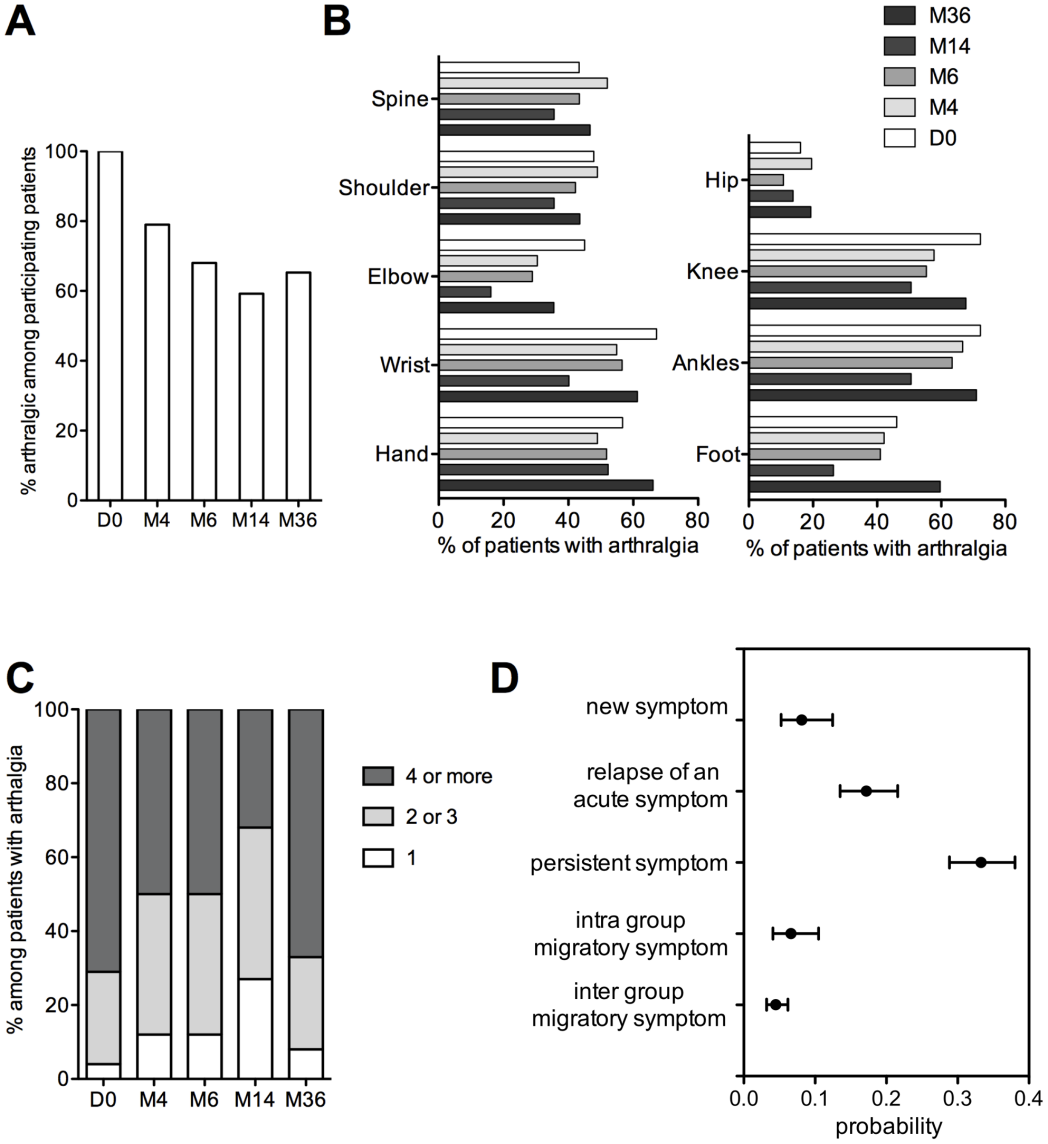
Evolution and characterization of arthralgia in CHIKV patients. (A) Percentage of patients with arthralgia among participating patients, (B) localization of arthralgia among patients with arthralgia, (C) number of arthralgia sites among the 76 patients answering at every time point and having arthralgia, (D) complete model presenting the probability for one joint to be painful depending on its previous state.

The percentage of patients suffering from long-term arthralgia decreased after CHIKV acute infection and stabilized around 60% (Figure 2A). Of note, all patients suffered from arthralgia at D0. Among them, only 5 on 180 (2.8%) suffered from joint pain prior to CHIKV infection.

Most patients had intermittent arthralgia, with recovery and relapse. For each time point, 25 to 40% of patients complained of permanent arthralgia. Among the 76 patients that could be followed at each time point, 45% had arthralgia at all time, 24% experienced partial recovery at M4, M6 or M14 then relapses, and 31% fully recovered from acute symptoms. Among patients who experienced chronic symptoms at M36, 43.5% reported arthralgia triggered by a change in ambient temperature, 8% by physical effort. At M36, arthralgia caused stiffness in 75.5% of patients with symptoms, and 67.7% of the patients reported a need of morning stretching (time of 32 minutes, standard deviation (SD) 37 minutes, range 5–180 minutes).

We monitored arthralgia in 9 anatomical sites (Figure 2B). Arthralgia in upper limbs mostly affected fingers and wrists, while lower limbs arthralgia mostly affected knees and ankles. At each time point, these locations remain significantly the most affected (Mac Nemar test for matched pairs of subjects). Importantly, arthralgia were typically symmetrical (90%).

We then investigated whether the number of arthralgic sites diminished in patients still suffering from arthralgia among the 76 patients followed at all time points. The number of arthralgia sites decreased until M14, with only 30% of patients suffering from polyarthralgia (number of arthralgia sites >2) (Figure 2C). Despite an increase of arthralgic sites at M36, there is an overall significant decrease of the number of painful joints during the study period (p<0.01).

We attempted to model the spatiotemporal evolution of arthralgia, as defined in the Materials and Methods section. We found that “persistent” symptoms had the strongest effect, as its probability of occurrence was three times higher than a “new” symptom (Figure 2D). The probability of relapse of an “acute” symptom was twice the appearance of a “new” symptom. Finally, “migratory” symptoms tended to be intra-group as compared to inter-group migrations.

### Other clinical signs associated with CHIKV chronic disease and their consequences

Patients with arthralgia at M36 showed other clinical symptoms, including local swelling, cutaneous symptoms, myalgia and osteoligamentaous pain (Table 1). Local swelling localized to affected joints for 63% of patients. Moreover, sleep, memory or concentration disorders and asthenia or depression are significantly associated with arthralgic patients.

**Table 1:** Clinical signs and treatment in arthralgic and non-arthralgic patients 36 months after the acute phase.

	Patients with arthralgia (n = 62)	Patients w/o arthralgia (n = 33)	Chi-2 test§
**Clinical signs**			
Local swelling	39 (62.9%)	0 (0%)	***
Osteo-ligamentous pain	22 (35.5%)	0 (0%)	***
Myalgia	24 (38.7%)	0 (0%)	***
Cutaneous lesion	31 (50.0%)	5 (15.1%)	***
Asthenia	48 (77.4%)	7 (21.2%)	***
Sleeping disorder	35 (56.4%)	3 (9.1%)	***
Dysgeusia	11 (17.7%)	3 (9.1%)	NS
Depression	31 (50.0%)	2 (6%)	***
Memory disorder	27 (43.5%)	2 (6%)	***
Concentration disorder	24 (38.7%)	2 (6%)	***
**Treatment**			
Followed by a general practioner	50 (80.6%)	1 (3%)	***
Treatment	51 (82.2%)	0 (0%)	***
Continous treatment	25 (49%)	0 (0%)	***
Paracetamol	45 (72.5%)	0 (0%)	***
Morphinic	0 (0%)	0 (0%)	
Non-steroidal anti-inflammatory drugs	12 (23.5%)	0 (0%)	**
Corticosteroids	3 (5.9%)	0 (0%)	NS

§ ns: Non significant.

** p<0.01.

*** p<0.001.

The proportion of patients with arthralgia who attended a physician or received a treatment significantly increased between M4 and M36 (p = 0.01) (data not shown), and reached 80% (Table 1). Similarly, the number of patients receiving a treatment increased and these treatments are statistically associated with the arthralgic status of the patient (p<0.001).

Arthralgia in patients at M36 were highly incapacitating for daily life tasks, professional life and spare-time activities (Table 2).

**Table 2:** Impact of arthralgia on daily life for patients at M36.

	Arthralgic patients (n = 62)	Non arthralagic patients (n = 33)	Chi-2 test§
**Impact on quality of life**			
rising from a chair	39 (48.4%)	0 (0%)	***
walking	34 (54.8%)	0 (0%)	***
picking up an object	34 (54.8%)	0 (0%)	***
opening a bottle	33 (53.2%)	0 (0%)	***
drinking glass	26 (41.9%)	0 (0%)	***
to wash oneself	23 (37.1%)	0 (0%)	***
At least one of these disabilities	48 (77.4%)	0 (0%)	***
**Impact on working life**			
With activity	15 (24.2%)	17 (51.5%)	
No impact	3 (20%)	16 (94.1%)	***
Physical impact	12 (80%)	1 (5.9%)	
low impact	7 (46.7%)	1 (5.9%)	
moderate impact	3 (20%)	0 (0%)	
high impact	2 (13.3%)	0 (0%)	
**Impact on leisure-time**			
No impact	13 (39.4%)	31 (93.9%)	
Physical impact	49 (79%)	2 (6.1%)	***
low impact	8 (24.2%)	1 (3%)	
moderate impact	18 (54.5%)	0 (0%)	
high impact	23 (69.7%)	1 (3%)	

§ **p<0.01. ***p<0.001.

### Identification of risk factors for developing long-term CHIKV-associated arthralgia

To identify risk factors associated with long-term arthralgia, we performed univariate and multivariate statistical analyses at M14, as the participation was higher than at M36 (Table 3). Gender was not associated with long-term arthralgia, age less than 35 years was protective. Risk of arthralgia was not associated with indicators of the disease severity during the acute phase (viral load, duration of hospitalization or number of sites of arthralgia at D0) [11], however it was weakly associated with C-reactive protein (CRP) level at D0. Diabetes was the only comorbidity found to be a risk factor for long-term arthralgia. Interestingly, arthralgia at M14 was strongly associated with arthralgia at M4, and even more if arthralgia was permanent at M4. Memory and concentration disorders at M4 were also identified as risk factors for developing long-term arthralgia. Arthralgia, memory disorders and concentration disorders at M4 were the only risk factors significantly and independently associated with long-term arthralgia. When long-term arthralgia was assessed at M36, results were very similar.

**Table 3:** Identification of risk factors for persistence of arthralgia 14 months after CHIKV acute disease (univariate analysis).

	Patients at M14 N	Patients with arthralgia N (%)	Odd Ratio (95% CI§)	p
Gender				
Male	73	41 (56.2%)	0.78 (0.40–1.51)	0.46
Female	74	46 (62.2%)	1	
Age (years)				
≤35	30	9 (30.0%)	0.18 (0.05–0.57)	**0.005**
36–50	28	19 (67.9%)	0.87 (0.26–2.84)	
51–60	24	17 (70.8%)	1	
61–70	20	15 (75.0%)	1.24 (0.32–4.73)	
>70	45	27 (60.0%)	0.62 (0.21–1.79)	
Hospitalization at D0				
No	101	61 (60.4)	1	0.66
Yes	46	26 (56.5)	0.85 (0.42–1.73)	
Viral quantification at D0 (copies/mL)				
≤100.000	70	39 (55.7)	1	0.41
>100.000	77	48 (62.3)	1.32 (0.68–2.54)	
CRP at D0 (unit)				
<10	20	7 (35.0)	1	**0.02**
>10	126	79 (62.7)	3.12 (1.16–8.38)	
Missing	1	1 (100.0)	No estimable	
Diabetes at D0				
No	109	58 (53.2)	1	**0.01**
Yes	38	29 (76.3)	2.83 (1.23–6.54)	
Arthralgia at M4				
No	26	5 (19.2)	**0.10 (0.04–0.30)**	**<0.001**
Yes	99	69 (60.7)	1	
Missing	22	13 (59.1)	0.63 (0.24–1.62)	
Arthralgia at M4				
No	26	5 (19.2)	**0.25 (0.08–0.78)**	**<0.001**
Yes. intermittent	43	21 (48.8)	1	
Yes. permanent	56	48 (85.7)	**6.28 (2.41–16.38)**	
Missing	22	13 (59.1)	1.51 (0.53–4.28)	
Memory disorder at M4				
No	87	41 (47.1)	1	**<0.001**
Yes	38	33 (86.8)	**7.40 (2.64–20.75)**	
Missing	22	13 (59.1)	1.62 (0.63–4.18)	
Concentration disorder at M4				
No	94	45 (47.9)	1	**<0.001**
Yes	31	29 (93.6)	**15.79 (3.56–69.97)**	
Missing	22	13 (59.1)	1.57 (0.61–4.03)	

§ confidence interval.

### Arthralgia status, systemic inflammation and autoimmunity markers

At M36, 22 patients with arthralgia (ART+) and 20 patients without arthralgia (ART−) were randomly selected from the cohort to participate to the CHIK IMMUNOPATH study. Its aim was to titrate anti-CHIKV antibodies and identify a serum inflammatory or autoimmune signature associated with the arthralgia phenotype.

All patients were negative for CHIKV RT-PCR, and exhibited anti-CHIKV IgGs in serum, while a minority (9.5%) harbored measurable levels of anti-CHIKV IgM. The activity of CHIKV IgG (GMAT) was significantly higher in ART+ patients (30) than in ART− patients (20), but antibody avidity was comparable in both groups (mean ±SD: 31,6±20,4 in ART + patients and 33,7±19,8 in ART− patients). Although lymphopenia is a defining feature of acute CHIKV disease [11], it was a rare finding at M36 (data not shown). Plasma protein levels measured by electrophoresis and CRP concentration were within normal ranges. However, CRP levels were significantly higher in the ART+ group than in the ART− group (mean ±SD: 3.35±3.00 mg/ml and 1.85±2.49 mg/ml, respectively; p = 0.04).

**Figure 3:**
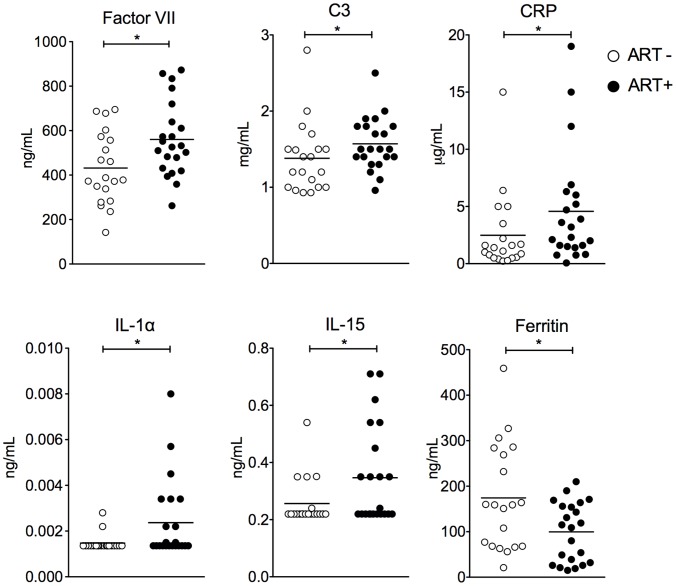
Distinctive markers in serum of patients with arthralgia (ART+ in black) versus patients without arthralgia (ART−, in white) at M36. SD: standard deviation, results of Mann Whitney test are shown. * p<0.05, q = 0.056.

We used Luminex xMAP technology to assay analytes in the serum of patients. Most analytes were undetectable in both groups of patients (Table 4). Five inflammation markers were significantly elevated in ART+ patients: factor VII, C3 complement component, IL1α, IL15 and CRP (Figure 3). Ferritin level was significantly lower in ART+ patients than in ART− patients. These markers did not allow for the identification of a subgroup within the ART+ group, nor did they correlate one with another. No autoimmune marker and no anti-DNA antibody in the serum of ART+ patients were detected, although anti-nuclear antibodies were detected at low level in four ART+ patients. Three patients had elevated anti-nuclear antibodies, one in the ART+ group and two in the ART- group.

**Table 4:** Analytes titrated in arthralgic and non arthralgic patients 36 months after the acute phase.

			Arthalgia (n = 20 patients)				No Arthalgia (n = 22 patients)				Statistic	
ATTRIBUTE	Units	LOD	DET	25%	MED	75%	DET	25%	MED	75%	M-W PV	M-W FDR
IP-10	pg/mL	116	18	170	214	376	20	126	207	379	0.537	0.606
Alpha-2-Macroglobulin A2Macro	mg/mL	0.472	18	0.6	0.7	0.9	21	0.7	0.8	1.1	0.194	0.434
Alpha-1-Antitrypsin AAT	mg/mL	1.33	20	1.9	2.1	2.6	22	1.8	2.0	2.1	0.305	0.606
Beta-2-Microglobulin B2M	ug/mL	0.914	20	1.1	1.4	1.9	22	1.2	1.3	1.6	0.481	0.606
Brain-Derived Neurotrophic Factor BDNF	ng/mL	4.7	20	12	17	24	22	14	18	21	0.940	0.966
Complement C3 C3	mg/mL	0.93	20	1.4	1.5	1.8	22	1.0	1.2	1.5	0.029	0.056
C-Reactive Protein CRP	ug/mL	0.063	20	1.5	2.8	5.8	22	0.5	1.3	2.4	0.035	0.056
Eotaxin-1	pg/mL	70.8	20	165	195	290	22	154	238	329	0.705	0.920
Factor VII	ng/mL	142	20	443	529	632	22	324	383	561	0.020	0.056
Fibrinogen	ug/mL	0.93	20	1.6	1.9	2.4	22	1.4	2.0	2.4	0.950	0.966
Ferritin FRTN	ng/mL	14.7	20	34	112	156	22	68	159	273	0.034	0.056
*Granulocyte-Macrophage Colony-Stimulating Factor*	*pg/mL*	*0*	*0*	*0.0*	*0.0*	*0.0*	*0*	*0.0*	*0.0*	*0.0*	*NA*	*NA*
Haptoglobin	mg/mL	0.007	20	1.0	1.6	2.1	21	0.6	0.9	1.8	0.194	0.434
Intercellular Adhesion Molecule 1 ICAM-1	ng/mL	32.1	20	88	98	119	22	87	107	121	0.801	0.920
*Interferon gamma IFN-gamma*	*pg/mL*	*0.9*	*6*	*0.9*	*0.9*	*0.9*	*2*	*0.9*	*0.9*	*1.0*	*0.038*	*0.056*
*Interleukin-1 alpha IL-1 alpha*	*pg/mL*	*1.51*	*2*	*1.4*	*1.4*	*3.1*	*10*	*1.4*	*1.4*	*1.4*	*0.007*	*0.010*
*Interleukin-1 beta IL-1 beta*	*pg/mL*	*2.68*	*0*	*2.4*	*2.4*	*2.4*	*2*	*2.4*	*2.4*	*2.4*	*0.091*	*0.112*
Interleukin-10 IL-10	pg/mL	1.55	17	1.6	2.2	2.8	18	1.6	2.2	2.6	0.808	0.920
*Interleukin-12 Subunit p40 IL-12p40*	*ng/mL*	*0*	*0*	*0.0*	*0.0*	*0.0*	*0*	*0.0*	*0.0*	*0.0*	*NA*	*NA*
*Interleukin-12 Subunit p70 IL-12p70*	*pg/mL*	*29.3*	*0*	*29*	*29*	*29*	*3*	*29*	*29*	*29*	*0.051*	*0.056*
*Interleukin-15 IL-15*	*ng/mL*	*0.2*	*5*	*0.2*	*0.2*	*0.4*	*11*	*0.2*	*0.2*	*0.2*	*0.021*	*0.056*
*Interleukin-17 IL-17*	*pg/mL*	*0.9*	*9*	*0.9*	*0.9*	*1.5*	*11*	*0.9*	*0.9*	*1.5*	*0.838*	*0.920*
Interleukin-18 IL-18	pg/mL	61.3	20	117	135	235	22	130	153	189	0.811	0.920
Interleukin-1 receptor antagonist IL-1ra	pg/mL	50.2	15	87	113	154	22	49	87	129	0.090	0.112
*Interleukin-2 IL-2*	*pg/mL*	*2.8*	*0*	*2.8*	*2.8*	*2.8*	*2*	*2.8*	*2.8*	*2.8*	*0.573*	*0.606*
*Interleukin-23 IL-23*	*ng/mL*	*0.4*	*8*	*0.4*	*0.4*	*0.7*	*8*	*0.4*	*0.4*	*0.7*	*0.787*	*0.920*
*Interleukin-3 IL-3*	*pg/mL*	*5.1*	*1*	*5.1*	*5.1*	*5.1*	*0.0*	*5.1*	*5.1*	*5.1*	*NA*	*NA*
*Interleukin-4 IL-4*	*pg/mL*	*2.6*	*1*	*2.6*	*2.6*	*2.6*	*5*	*2.6*	*2.6*	*2.6*	*0.111*	*0.112*
Interleukin-5 IL-5	pg/mL	3.8	11	3.8	4.8	6.8	16	3.4	3.8	5.1	0.168	0.406
*Interleukin-6 IL-6*	*pg/mL*	*5.8*	*1*	*5.8*	*5.8*	*5.8*	*0*	*5.8*	*5.8*	*5.8*	*0.133*	*0.352*
*Interleukin-7 IL-7*	*pg/mL*	*5*	*4*	*5.0*	*5.0*	*5.0*	*1*	*5.0*	*5.0*	*5.0*	*0.112*	*0.112*
Interleukin-8 IL-8	pg/mL	2.8	19	9	17	46	20	9	11	24	0.650	0.897
Monocyte Chemotactic Protein 1 MCP-1	pg/mL	33.7	20	105	170	216	22	129	150	172	0.435	0.606
Macrophage Inflammatory Protein-1 alpha MIP-1 alpha	pg/mL	10.6	15	11	16	46	16	11	27	36	0.869	0.933
Macrophage Inflammatory Protein-1 beta MIP-1 beta	pg/mL	112	20	194	284	431	22	242	269	328	0.950	0.966
*Matrix Metalloproteinase-2 MMP-2*	*ng/mL*	*0*	*0*	*0.0*	*0.0*	*0.0*	*0*	*0.0*	*0.0*	*0.0*	*NA*	*NA*
Matrix Metalloproteinase-3 MMP-3	ng/mL	2.31	20	2.9	5.7	8.8	22	5.5	6.9	10.3	0.162	0.406
Matrix Metalloproteinase-9 MMP-9	ng/mL	1.3	12	1.2	1.3	1.3	15	1.2	1.3	1.3	0.305	0.606
T-Cell-Specific Protein RANTES RANTES	ng/mL	3.81	20	7	16	21	22	9	17	23	0.345	0.606
Stem Cell Factor SCF	pg/mL	60.4	20	155	192	229	22	140	167	219	0.239	0.514
Tissue Inhibitor of Metalloproteinases 1 TIMP-1	ng/mL	68.7	20	120	132	169	22	126	144	177	0.623	0.897
Tumor Necrosis Factor alpha TNF-alpha	pg/mL	1.33	20	2.0	2.2	2.7	21	1.8	2.2	2.7	0.643	0.897
*Tumor Necrosis Factor beta TNF-beta*	*pg/mL*	*12.1*	*1*	*12*	*12*	*12*	*1*	*12.1*	*12.1*	*12.1*	*0.612*	*0.897*
Tumor Necrosis Factor Receptor-Like 2 TNFR2	ng/mL	2.51	20	3	4	8	22	3.3	4.0	5.2	0.364	0.606
Vascular Cell Adhesion Molecule-1 VCAM-1	ng/mL	323	20	460	595	707	22	490	539	640	0.606	0.606
Vitamin D-Binding Protein VDBP	ug/mL	114	20	158	273	372	22	237	259	303	0.762	0.920
Vascular Endothelial Growth Factor VEGF	pg/mL	288	20	458	628	763	22	492	579	786	0.811	0.920
von Willebrand Factor vWF	ug/mL	13.7	20	21	28	35	22	20	26	34	0.762	0.920

DET: number of patients with detectable value within the group.

MED: 25%; 75%:median and interquartile.

LOD: lowest limit of detection.

M-W PV: Mann Whithney P value.

M-W FDR Mann Whitney with False Discovery Rate Correction.

As it has been reported that CHIKV could evolve into rheumatoid arthritis [15], we screened for cyclic citrullinated protein antibodies. We also assayed for cryoglobulinemia and anti-endomysium IgA antibodies. All patient were found to be negative.

### Estimation of the economic impact of CHIKV long-term arthralgia

We estimated the annual economic burden of long-term arthralgia by taking into account the cost of medical visits, therapeutic treatment and the cost for lost work time due to injury or pain (using the population of La Réunion Island as a reference) (Table S2). We found that arthralgia secondary to the CHIKV outbreak in La Réunion in 2005-06 has resulted so far in an estimated total cost of up to 34 millions euros per year. This corresponds to 250€ per year and per patient with long-term arthralgia. However, it should be noted that this sum might be overestimated due to the bias in our cohort selection, as our cohort is likely composed of the most severely affected patients who were referred to the hospital during the acute phase.

## Discussion

Our study is the first prospective cohort study on CHIKV long-term arthralgia that is based on the follow-up of patients who presented with acute CHIKV infection as the inclusion criterion. This study is also the first to define the evolution of CHIKV-induced arthralgia, mapping the frequency and location of arthralgic sites during a three year time period. We have also investigated the impact of CHIKV-chronic arthralgia on daily life of patients, identified clinical signs associated with arthralgia, and analyze biologic markers. Moreover we have evaluated associated risk factors and estimated the economic burden of this disease. Together, these data allow us to define the features of CHIKV-induced chronic arthralgia (Table 5), as compared to other viral arthritis [16], and to establish a detailed understanding of the public health problem resulting from CHIKV-chronic arthralgia.

**Table 5:** Features of patients with chronic CHIKV-associated arthralgia.

Age	>35
Sex ratio (M/F)	1/1
Number of sites	oligo or polyarthralgia
Sites	upper limbs : fingers. wrist
	lower limbs : knees. ankles
Type	symmetrical
	permanent or not
	migratory
	highly incapacitating
	morning stiffness duration average: ≈30 minutes
General sign fever	no fever
Other clinical signs	edema
	cutaneous lesion
	myalgia
	sleep and memory disorders
Laboratory tests	CRP normal
	ACCP normal
	antinuclear normal
	anti CHIKV IgGs positive
Risk factor	diabetes

Our data reveal that more than 60% of CHIKV-infected patients suffer from arthralgia, 36 months after acute infection. This high percentage of patients with long-term symptoms was also reported by other studies of Italian cohorts and French cohorts of La Réunion Island or metropolitan France [17], [18], [19], [20], [21] but is dramatically higher than documented in India and Senegal [1], [22], [23], [24], [25]. While this discrepancy may result from particular features of the CHIKV strain responsible for the La Réunion outbreak, data from Italy following the 2007 outbreak resulted from a CHIKV strain more closely related to the viral strain present in India [10], with more than 60% of CHIKV patients in Italy having reported myalgia, asthenia or arthralgia 12–13 months after the acute disease [20]. Alternatively, reported differences may be a result of different genetic backgrounds of these populations. As joint pain is considered a subjective symptom, it might also reflect a difference in pain threshold of patients or reporting from physicians, thus reflecting differences in health care practices.

Long-term CHIKV-associated arthralgia were mainly symmetrical, involving more than 2 different joints. Hand, wrist, ankle and knee were found to be the most affected, consistent with other studies [17], [18]. Moreover, 60–80% of patients had relapsing arthralgia, while 20–40% had unremitting arthralgia. While some patients reported “migrating” arthralgia, most disease symptoms mapped to joints that were most painful during acute Chikungunya disease. Thus, symptoms at the chronic phase may be indirectly associated to virus replication at the time of acute infection [26], [27], [28].

In addition to arthralgia, many patients suffered from myalgia and cutaneous lesions and several cognitive dysfunctions. Although study patients did not display neurological symptoms at the acute phase of disease, we cannot exclude that cognitive dysfunctions result from CHIKV spread in the CNS, as it has been reported that CHIKV disseminates to the CNS in humans and in animal models [12], [26], [27], [29]. Similar to other studies, chronic arthralgia are considered incapacitating for daily life tasks and impacted professional activities and quality of life [20], [21]. Beside this impact on patient, the economic burden of this long-term pathology is also very significant, independently of the cost of the acute disease [30].

The longitudinal design of our study enabled us to identify risk factors for development of long-term arthralgia. Individuals over the age of 35 years or with diabetes were more likely to suffer from chronic arthralgia. The age has been reported to be a risk factor with some cohorts [21], [31], [32], but not in others [18], [33]. None of our available parameters to measure the severity of acute disease were associated with long-term arthralgia. This may be explained by differences in the way to measure disease severity in other studies [18]. Importantly, we show that the presence and intensity of arthralgia at M4 after the onset of the acute disease is a good predictor of long-term arthralgia.

Our study did not identify positive markers for autoimmune or rheumatoid arthritis. Additionally, we failed to identify systemic biomarkers associated with the arthralgic phenotype. Nevertheless, a slightly more elevated inflammatory status is found in a subset of arthralgic patients who have detectable serum level of IL1α, IL15 and slight elevation in Factor VII, C3 and CRP. This signature differs from that observed at the onset of the infection, when circulating virus is detectable and type I interferon, IP10, MCP1, ISG15 are highly elevated [34], [35], [36]. Others have identified IL6 and GM-CSF or IL12 as being specifically associated with long-term arthralgia [32], [33]. However, these studies were performed much earlier in the chronic phase (2–3 months and one year after disease onset).

Our study shows that anti-CHIKV antibody titers were more elevated in ART+ patients than in ART- patients. This is in agreement with a recent study [37]. This higher level of antibodies could be associated with a more severe acute infection [37]. However, in our study, the level of antibody at M36 did not correlate with acute disease severity. Alternatively, this could reflect a persisting antigenic stimulation in ART+ patients (see below). Interestingly, it has been reported that viremia level at the acute phase correlates with a faster appearance of neutralizing antibodies and a better recovery 2–3 months after the acute phase [38].

Similarly to CHIKV, other so called “arthritogenic” alphaviruses, notably Ross River virus (RRV), are known to cause acute as well as chronic arthralgia [39]. Our data indicates that chronic symptoms are linked to the initial local joint inflammation and are not associated with markers of systemic inflammation or autoimmunity. A local inflammation of the joint could be maintained by the local persistence or delayed clearance of viral antigens. This is consistent with report of Hoarau *et al*. [32] who detected persistent CHIKV antigens within the synovial fluid of a patient suffering from chronic arthralgia. Moreover, experimental studies in CHIKV infected animal indicate that the joint is the most highly infected tissue, making it plausible that incomplete viral antigen clearance in this anatomical site may account for the long-term symptoms [26]. RRV has been shown to persist *in vitro* in mouse macrophages, and a model of RRV chronic arthritis suggests that viral persistence may account for chronic disease [40]. RRV and CHIKV have been shown to be weakly tropic for macrophages *in vitro* [41], [42] but the presence of antibodies dramatically increased RRV entry into macrophage [42]. Further studies will be required to assess the role of macrophages, as well the role of persistent infection and antibodies in chronic arthritis caused by CHIKV.

In sum, this study furthers our understanding of the pathophysiology of CHIKV chronic arthralgia, a prerequisite for the development of efficient therapeutic strategies and for assessing the burden of disease inflicted upon populations affected by epidemic Chikungunya disease.

## Supporting Information

Supporting Information S1 Strobe checklist for cohort study.
(DOC)

Supporting Information S2 Questionnaire used for the study.
(DOCX)

Supporting Information S3 Modelisation and estimation of spatiotemporal dynamics of arthralgia.
(DOC)

Table S1 Likelihood ratio tests testing the complete model against different submodels.
(DOC)

Table S2 Estimation of the annual economic impact of CHIKV long term arthralgia.
(DOC)
