# Correction: Chikungunya Virus-associated Long-term Arthralgia: A 36-month Prospective Longitudinal Study

**DOI:** 10.1371/annotation/850ee20f-2641-46ac-b0c6-ef4ae79b6de6

**Published:** 2013-03-22

**Authors:** Clémentine Schilte, Frédérik Staikovsky, Thérèse Couderc, Yoann Madec, Florence Carpentier, Somar Kassab, Matthew L. Albert, Marc Lecuit, Alain Michault

The second author's name was misspelled. The correct name is Frederik Staikowsky.

The correct citation is: Schilte C, Staikowsky F, Couderc T, Madec Y, Carpentier F, et al. (2013) Chikungunya Virus-associated Long-term Arthralgia: A 36-month Prospective Longitudinal Study. PLoS Negl Trop Dis 7(3): e2137. doi:10.1371/journal.pntd.0002137

