# Supplementary material for: Chikungunya Virus-associated Long-term Arthralgia: A 36-month Prospective Longitudinal Study
Source: PLoS Negl Trop Dis. 2013 Mar 21;7(3):e2137. doi: 10.1371/journal.pntd.0002137 (PMC3605278; doi:10.1371/journal.pntd.0002137)
Supplement: Supporting Information S2 — Questionnaire used for the study. (DOCX) [file pntd.0002137.s002.docx]

**Questionnaire**

Phone call date …/…. /…..

Contacted

Not contacted

Other non inclusion criteria than « not contacted » :

Death

Do not want to participate

Other. : precise…….

1. Since your infection with chikungunya, are your joint still painful?

Yes  No

- 1. Which joints are painfull?

|  | right | left |
| --- | --- | --- |
| Finger |  |  |
| Wrist |  |  |
| Elbow |  |  |
| Shoulder |  |  |
| Hip |  |  |
| Knee |  |  |
| Ankle |  |  |
| Foot |  |  |
| Rachis |  |  |

- 1. Are your joint pain:

Permanent  Yes  No

Intermittent  Yes  No

Do they wake you up during nights?  Yes  No

1. Do you experience morning stiffness?  Yes  No
   1. Do you require a stretching time in the morning?  Yes  No

For how long? ………. minutes

1. Are some factors triggering or raising up your joint pain?  Yes  No

If yes, precise: ………………….

1. Do you have persisting oedema at the joint site?
2. Do you still have some troubles accomplishing the following tasks:

| raising from a chair | Yes | No |
| --- | --- | --- |
| walking | Yes | No |
| picking up an object | Yes | No |
| opening a bottle | Yes | No |
| drinking glass | Yes | No |
| to wash oneself | Yes | No |

1. Do your tendons or bones hurt?
2. Do your muscles still hurts?
3. Do you still have skin abnormalities since you Chikungunya virus infection?

If yes: precise : ……….

1. Do you suffer from fatigue?  Yes  No
2. Do you sleep well?  Yes  No
3. Since your infection do you have a taste disorder?  Yes  No
4. Do you have memory disorder since CHIKV disease?  Yes  No
5. Did you have concentration disorder since CHIKV disease?  Yes  No
6. Do you have a professional activity?

If yes, what impact has your disease on your professional activity

null

low

medium

high

1. Is there an impact on your leisure time

null

low

medium

high

1. Are you attending a physician because of your chikungunya disease?
2. Do you take medecin because of chikungunya disease?

Every day?

| If yes which one : |  |  |
| --- | --- | --- |
| Paracetamol | Yes | No |
| Corticoid | Yes | No |
| Morphine | Yes | No |
| Nivaquine | Yes | No |
| Antidepressant | Yes | No |
| Other: precise……….…………….. | | |

1. Did you had arthralgia before your chikungunya disease?

If yes precise: ……………….
