# Supplementary material for: Chikungunya Virus-associated Long-term Arthralgia: A 36-month Prospective Longitudinal Study
Source: PLoS Negl Trop Dis. 2013 Mar 21;7(3):e2137. doi: 10.1371/journal.pntd.0002137 (PMC3605278; doi:10.1371/journal.pntd.0002137)
Supplement: Supporting Information S3 — Modelisation and estimation of spatiotemporal dynamics of arthralgia. (DOC) [file pntd.0002137.s003.doc]

# Modelisation and estimation of spatiotemporal dynamics of arthralgia

The model of spatio-temporal dynamics of arthralgia describes *Y_i,j,t_*, the state of a site *j* of the individual *i* at the survey *t*. *Y_i,j,t_* is coded in 1/0 i.e. painful/not painful. This model is dynamic, more precisely takes into account the states of the sites of the individual *j* at the precedent survey *t-1* and during the acute phase *t_0_*. The apparition of arthralgia is supposed to come from four different and independent processes: “persistent symptom”, "relapse of an acute symptom ", "new symptom" and "migration symptom".


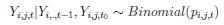


with *J-{j}*, the set of sites observed except the site *j* and *Y_i,.,t_* is the set *{Y_i,j,t_ ; j ∈ J}*. Process probabilities are described by the parameters *α, β_0_, β_1_, γ_w_* and *γ_b_*, whose values belong to the interval [0,1].

with *Gj*, the set of the sites belonging to the group of the site *j* (top, middle or down).

The state *Y_i,j,t_* are assumed to be independent conditionally to the states of the sites of the individual *j* at the precedent survey *t-1* and during the acute phase *t_0_*. Thus the model parameters were obtained by maximizing the following likelihood:

*Tj* is the set of survey where the individual *j* has been observed, excluding the survey whose precedent survey was unknown and thus, excluding at least the acute phase (*t_0_*).

The effects of the different processes were tested using likelihood ratio tests comparing this (complete) model against the null hypothesis model (same probability of arthralgia appearing in all sites and at all times i.e. only “new symptom”) and against three sub-models. The first sub-model considers no “persistent symptom” and it is used to test the “persistent symptom” effect. The second sub-model considers that the probability of apparition of arthralgia not due to a “persistent symptom” or a “migration symptom” is constant and thus not modified by an affection of the site at the acute phase. This means that the probability of “a relapse of an the acute symptom” is equal to those of “a new symptom”. It is used to test the effect of the “a relapse of an acute symptom”. The third sub-model considers no “migration symptom” and it is used to test the “migration symptom”.

These statistical analyses were performed in R (CRAN-Project).
