# Supplementary material for: Chikungunya Virus-associated Long-term Arthralgia: A 36-month Prospective Longitudinal Study
Source: PLoS Negl Trop Dis. 2013 Mar 21;7(3):e2137. doi: 10.1371/journal.pntd.0002137 (PMC3605278; doi:10.1371/journal.pntd.0002137)
Supplement: Table S1 — Likelihood ratio tests testing the complete model against different submodels. (DOC) [file pntd.0002137.s004.doc]

**Table S1.** Likehood ratio tests testing the complete model against different submodels

| **Test** | **Constraints of submodel parameters** | **p value** |
| --- | --- | --- |
| Null model | β_0_=β_1_, α=γ_ω_=γ_b_=0 | 7.7 10-91 |
| "continuation" effect test | α=0 | 4.8 10-37 |
| "spontaneous apparition" effect test | β_0_=β_1_ | 1.7 10-5 |
| "migration" effect test | γ_w_=γ_b_=0 | 6.7 10-15 |
