# Supplementary material for: Chikungunya Virus-associated Long-term Arthralgia: A 36-month Prospective Longitudinal Study
Source: PLoS Negl Trop Dis. 2013 Mar 21;7(3):e2137. doi: 10.1371/journal.pntd.0002137 (PMC3605278; doi:10.1371/journal.pntd.0002137)
Supplement: Table S2 — Estimation of the annual economic impact of CHIKV long term arthralgia. (DOC) [file pntd.0002137.s005.doc]

**Table S2**. Estimation of the annual economic impact of CHIKV long term arthralgia

| **Population** | |  |  |  |
| --- | --- | --- | --- | --- |
| La Réunion inhabitants | | 784,000 |  | persons |
|  | 73% older than 15 | 573,992 |  | persons |
|  | 38% seroprevalence | 218,117 |  | persons |
|  | 95% symptomatic patients | 207,000 |  | persons |
|  | 65% arthralgic at 36 months | 135,000 |  | persons |
|  |  |  |  |  |
| **Cost of medical visit** | |  |  |  |
|  | 49% patients with a continous treatment | 60,615 |  | persons |
|  | number of medical visit/year | 2 |  |  |
|  | 51% patients with a discontinous treatment | 66,150 |  | persons |
|  | number of medical visit/year | 1 |  |  |
|  | medical visit price in France | 23 |  | € |
|  |  | **Total** |  | **4,6 M€** |
| **Drug cost** | |  |  |  |
| 82% following a treatment at M36 | | 111 000 |  | persons |
|  | 49% patients with a continous tratment |  |  |  |
|  | Days of treatment/ year | 365 |  | days/year |
|  | paracetamol (3g/days) | 0,795 |  | €/day |
|  | 51% patients with a discontinous tratment |  |  |  |
|  | Days of treatment/ year | 30 |  | days/year |
|  | paracetamol (3g/days) | 0,795 |  | €/day |
|  | Nonsteroidien antininflammatory drugs (200 mg/days) | 0,493 |  | €/day |
|  |  | **Total** |  | **18 M€** |
| **Cost of lost work** | |  |  |  |
| 21% having a professional activity at M36 | | 28 350 |  | persons |
|  | 13% with high impact |  |  |  |
|  | estimated decrease of activity | 10% |  | % |
|  | 20% with moderate impact |  |  |  |
|  | estimated decrease of activity | 5% |  | % |
|  | Minimal annual french income | 16380 |  | € |
|  |  | **Total** |  | **11 M€** |
|  |  |  |  |  |
| **Overall cost** | |  |  | **34 M€** |
